# Item-dependent cues in serial order are tracked by the magnitude (not the presence) of the fill-in tendency

**DOI:** 10.3758/s13423-025-02684-8

**Published:** 2025-04-08

**Authors:** Dakota R. B. Lindsey, Tyler L. Harrison

**Affiliations:** 1 Department of Psychology, University of South Alabama, 307 N. University Blvd., Mobile, AL 36688 USA; 2 University of North Georgia, Dahlonega, USA

**Keywords:** Fill-in, Serial recall, Serial learning, Typing, Serial order

## Abstract

In tasks that measure serial-order memory, it is common to observe a “fill-in tendency”—when a person skips an item, the next item they report is more likely to be the skipped item (a fill-in response) than the next list item (an infill response). They tend to “fill in” the blank they skipped. The fill-in tendency has informed the modeling of serial-order memory—it presents strong evidence against associative chaining accounts because they predict more infill responses than fill-in responses. Despite the failures of associative chaining theories, evidence grows for the use of chaining-like item-dependent cues in serial-order memory. In this paper, we analyzed fill-in and infill responses from nine serial learning experiments (one new experiment and eight previously published experiments) that used variants of the spin list procedure and found strong evidence of item-dependent retrieval cues in serial-order memory. The current analyses revealed a fill-in tendency in all lists—even in those in which item-dependent cues were suspected to have been used. However, in those lists the likelihood of infill responses was higher, and consequently, the fill-in tendency was weaker. Our results expose a flaw in the conventional understanding of fill-in and infill responses. That is, the presence (or absence) of the fill-in tendency is not a strong test of item-dependent cues. Instead, changes in the magnitude of the fill-in tendency—more specifically, an increase in the likelihood of infill responses—across task conditions seem to better indicate the use of item-dependent cues.

**Supplementary Information:**

The online version contains supplementary material available at 10.3758/s13423-025-02684-8.

Serial-order memory supports a vast array of real-world behaviors, including typing (Logan, 2018), playing music (Pfordresher et al., 2007), reading (Ordonez Magro et al., 2020), spelling (Houghton, 2018), speech (Dell et al., 1997), and route navigation (Hilton et al., 2021). Many researchers have studied the mechanisms that support recall of information in order (e.g., see, Hurlstone, 2024; Hurlstone et al., 2014; Logan, 2021). Often, these studies employ a *serial recall* procedure, in which researchers present a list of items and ask participants to recall those items in their presented order (e.g., Conrad, 1964), or a *serial learning* procedure, in which researchers ask participants to repeatedly recall lists of items in order (e.g., Hebb, 1961). Over a century’s worth of research has produced numerous benchmark phenomena that constrain theories of serial-order memory (Oberauer et al., 2018). The current paper focuses on just one of these benchmarks: the tendency for participants to go back to “fill-in” an item they previously skipped (e.g., Henson, 1996).

In serial-order tasks, participants occasionally commit *anticipations*, in which they report a list item too early (e.g., reporting ABD to the list ABCDEF). After anticipating, participants often move backward in the list to report a skipped item (ABDC) or continue forward to the next item (ABDE). Moving backward to report a skipped item is a pattern known as *fill-in*—the participant “fills-in” the skipped item. Moving forward to the next item is conversely known as *infill*. Fill-in is often more likely than infill—this is a benchmark of serial order (Oberauer et al., 2018; Osth & Hurlstone, 2023)^1^ known as the *fill-in tendency* (Norris et al., 1994). A 2:1 ratio of fill-in to infill responses is often reported (e.g., Surprenant et al., 2005).

The fill-in tendency is observed in serial recall tasks (Farrell et al., 2013; Henson, 1996; Osth & Dennis, 2015), serial reconstruction of order tasks (Osth & Dennis, 2013; Surprenant et al., 2005), and copy typing tasks (Logan, 2021) when list length is short (around five items). It is not observed in serial recall tasks that present long lists of items (Farrell et al., 2013; Solway et al., 2012) or that sample list items from a large open set of items instead of a small closed set (Osth & Dennis, 2013, 2015). In these scenarios, a reverse fill-in tendency, or *infill tendency*, is more common; participants are more likely to continue forward in the list.

The fill-in tendency played an important role in the demise of *associative chaining* theories of serial order. Chaining theories claim that associations are formed between each item and that previously recalled items serve as cues to retrieve subsequent items from memory. In classical chaining models (e.g., Lashley, 1951) there are only forward associations, so it is not possible to move backward to fill-in skipped items. More recent chaining theories allow backward associations but assume that forward associations are stronger (Solway et al., 2012) or that equal-strength backward associations are suppressed after response (Caplan, 2015; Lewandowsky & Murdock, 1989). In the former case, an anticipated item most strongly cues the next list item, producing an infill tendency. In the latter, the anticipated item cues the preceding item and subsequent item equally, producing roughly equal infill and fill-in. These predictions do not align with the fill-in tendency, so it presents strong evidence against chaining accounts.

Favor has shifted from chaining theories to those that use retrieval mechanisms that operate independently of previously retrieved items—often by cuing memory with representations of serial position (e.g., Brown et al., 2000; Henson, 1996; Lewandowsky & Farrell, 2008) that we call *item-independent cues*. With the support of ancillary assumptions—such as a primacy gradient that more strongly activates early list items (Henson, 1996)—theories that use item-independent cues can produce a fill-in tendency. Despite the theoretical shift, empirical support continues to grow for a chaining-like mechanism (Caplan, 2015; Cowan & Hardman, 2021; Fischer-Baum & McCloskey, 2015; Kahana et al., 2010; Lindsey & Logan, 2019, 2021; Solway et al., 2012). In addition to item-dependent cues, serial retrieval might be supported by the most recently recalled item (Solway et al., 2012), several previously recalled items (Murdock, 1995), or contextual states that are related to recalled items (Logan, 2021). We call these representations *item-dependent cues*. Collectively, the evidence is most consistent with *hybrid theories* that incorporate both item-dependent cues and item-independent cues—it does not support a strict chaining (or serial position coding) account.

The spin list procedure—a serial learning task in which participants practice recalling rotated lists called *spun lists* (e.g., ABCDEF, FABCDE, EFABCD)—is noteworthy because it has been used to support both item-independent cues (Ebenholtz, 1963) and item-dependent cues (Kahana et al., 2010; Lindsey & Logan, 2019, 2021). In spun lists, each item has consistent neighbors (B is always after A) but appears in each serial position (B can be first, second, etc.). Comparing spun lists to *same lists*—in which items are shown in the same serial positions on each presentation (ABCDEF, ABCDEF)—reveals that learning is slowed in spun lists where serial position is inconsistent. The *same list advantage* (i.e., higher recall accuracy for same lists than spun lists) is consistent with the use of item-independent cues; position-based cues can support the learning of same lists but not spun lists. Comparing spun lists to *scrambled lists*—in which items appear equally often in each serial position and next to each other item (ABFCED, BCADFE, CDBEAF)—reveals that learning is slowed in scrambled lists where neighboring items are inconsistent. The *spun list advantage* (i.e., higher recall accuracy for spun lists than scrambled lists) is consistent with the use of item-dependent cues; item-based cues can support the learning of spun lists, but not scrambled lists. Table 1 shows examples of each list type.

**Table 1:** Construction of same, spun, and scrambled lists

	Same set						Spun set						Scrambled set					
List 1	A	B	C	D	E	F	G	H	I	J	K	L	M	N	O	P	Q	R
List 2	A	B	C	D	E	F	L	G	H	I	J	K	N	P	M	R	O	Q
List 3	A	B	C	D	E	F	K	L	G	H	I	J	P	R	N	Q	M	O
List 4	A	B	C	D	E	F	J	K	L	G	H	I	R	Q	P	O	N	M
List 5	A	B	C	D	E	F	I	J	K	L	G	H	Q	O	R	M	P	N
List 6	A	B	C	D	E	F	H	I	J	K	L	G	O	M	Q	N	R	P

*Note.* The letters in this table represent different items. In same lists, the order of presentation was always the same. In spun lists, the order of presentation was rotated. In scrambled lists, the order of presentation was scrambled using a balanced Latin square. In the current experiments, lists were constructed from nonoverlapping sets of six letters, and the letters in each set were selected randomly for each participant

The spun list advantage reflects an increased reliance upon item-dependent cues in spun lists. Because associative chaining theories (the most tested implementation of these cues) predict an infill tendency, one might anticipate an infill tendency in spun lists. However, a comprehensive analysis of fill-in and infill has so far not been conducted for spin list procedures. The current paper fills this gap by reporting analyses of fill-in and infill in nine spin list experiments. Eight of these have already been published (Lindsey & Logan, 2019, 2021). One is new to this paper. The published experiments compare spun and scrambled lists. The new experiment compares same, spun, and scrambled lists.

Following the predictions of chaining theories, we expect to observe an infill tendency in spun lists. Scrambled list recall mirrors standard serial recall tasks in which participants recall short lists of randomly rearranged items. Like those tasks, we expect to observe a fill-in tendency in scrambled lists. Same list recall mirrors recall of repeated lists in Hebb repetition tasks, for which chaining accounts have also been rejected (e.g., Hitch et al., 2005). We also expect to observe a fill-in tendency in same lists. To preface the results, we found a fill-in tendency for all list types. However, the magnitude of the fill-in tendency was weaker in same lists and spun lists—where item-dependent cues can support learning. The analyses reveal misconceptions about the fill-in tendency and serial learning.

## Method

Brief methodological descriptions of the experiments are provided below. We first summarize the procedures and key findings reported by Lindsey and Logan (2019, 2021). Refer to the original papers for additional information. Afterward, we summarize the procedures and key findings of a new experiment. Detailed methods and results for this experiment can be found in Appendix A in the supplemental materials. The main body of the paper focuses on analyses of fill-in and infill in the analyzed experiments.

### Lindsey and Logan (2019)

Lindsey and Logan (2019) compared spun and scrambled lists in copy typing. They recruited 24 skilled typists for each experiment. Participants were shown six-letter lists, which appeared on the screen simultaneously in a row. Participants were asked to type the lists as quickly and accurately as they could in left-to-right order. The letters were visible while participants typed their responses, so explicit memory of the list was not required to complete the task. Half of the typed lists were spun lists, and the other half were scrambled lists. Participants practiced typing each spun list and each scrambled list 40 times. The timing and accuracy of each keystroke was recorded. Because accuracy was near ceiling, their analyses focused on typing speed in each of the list types.

We analyzed data from Experiments 1, 2, and 4. Experiments 2 and 4 included additional transfer trials at the end of the task, but these transfer trials are not included in the present analyses. Experiment 3 was excluded from analysis because it did not include a scrambled list comparison condition. A spun list advantage was observed in the data for each analyzed experiment: Average keystroke times were shorter for spun lists than scrambled lists, indicating that item-dependent cues aided the typing of spun lists.

### Lindsey and Logan (2021)

Lindsey and Logan (2021) compared spun and scrambled lists in serial recall. They recruited 24 participants for each experiment. The experiments shared many similarities with the Lindsey and Logan (2019) typing experiments. Participants saw a list of six letters, presented simultaneously in a row. The list disappeared, and after a brief retention interval the participants were asked to type the letters they could remember in left-to-right order. Participants practiced recalling each spun and list and each scrambled list 40 times. The timing and accuracy of each response was recorded, and analyses focused on the frequency and type of errors committed in spun and scrambled lists.

We analyzed data from Experiments 1, 2, 3, 4, and 5. Experiment 6 was excluded because it included several methodological changes that made it distinct from the other experiments (e.g., participants recalled sequentially presented lists of words instead of simultaneously presented lists of letters). Experiments 2, 3, 4, and 5 included transfer trials that are not included in the present analyses. A spun list advantage was observed in the analyzed data for each of the experiments^2^: Average recall accuracy was higher for spun lists than scrambled lists. Item-dependent cues aided the recall of spun lists. The spun list advantage was specific to order errors, indicating that these cues helped participants keep list items in order.

### New experiment

In the new experiment, we compared same lists, spun lists, and scrambled lists in serial recall. Participants practiced recalling each list type 10 times each. We reduced the number of repetitions to shorten the time to complete the task—most of the learning in prior iterations of this procedure (Lindsey & Logan, 2021) occurred within the first 10 repetitions of each list. The task procedure was otherwise the same as Lindsey and Logan: each list was six letters in length, the letters were presented simultaneously for 500 ms, and participants recalled them in left-to-right order after a brief retention interval of 500 ms. We collected accuracy and response time data from 223 participants across two sites (University of South Alabama and University of North Georgia).

The results were consistent with previously published research. There was a same list advantage (cf. Kahana et al., 2010) and a spun list advantage (cf. Lindsey & Logan, 2021): Error rates were lower in same lists than spun lists, and lower in spun lists than scrambled lists. All types of error were less likely in same lists than in spun lists. Only order errors were less likely in spun lists than scrambled lists. The results support the use of both item-dependent and item-independent cues by serial memory.

### Lag-conditional response probability (Lag-CRP) curves

Following other recent analyses of the fill-in tendency (Farrell et al., 2013; Osth & Dennis, 2015; Solway et al., 2012), we assessed the likelihood of responses by computing *lag-conditional response probability* (lag-CRP). Lag-CRP is the likelihood of making a recall transition of a particular distance, or *lag*, from the previously recalled item. Consider the input list ABCDEF and the output list ABDC. The transition from A to B in the output list is a + 1 lag transition because the recalled Item B appeared one position after A in the input list. The transition from B to D is a + 2 lag transition because D appeared two positions after B in the input list. The transition from D to C is a − 1 lag transition because C appeared one position before D in the input list. Probabilities are computed for each lag by dividing the number of times a lag occurred (e.g., a − 1 lag occurred only once in the output list) by the number of opportunities for that lag to occur (e.g., a − 1 lag was possible after each item, except A because it was the first list item). As such, the lag-CRP accounts for differences in the frequencies of response transitions that might occur simply because of list constraints (e.g., there are more opportunities to make short-lag transitions than long-lag transitions).

Fill-in responses involve backward movement in the list, usually producing − 1 lag responses. Infill responses involve forward movement in the list, usually producing + 1 lag responses. However, a repeat of a previously recalled item (e.g., ABA) can also produce a − 1 lag, and most correct responses (ABA) produce + 1 lags. When all responses are included in lag-CRP computations, it is not possible to assess the frequency of fill-in or infill. To circumvent this issue, lag-CRP calculations are further conditionalized to include only responses given after an anticipation error (Solway et al., 2012). In the output list ABDC, response D represents an anticipation error because it was reported too early. Only the following response, C, would contribute to the postanticipation lag-CRP. Response C has a − 1 lag, indicating that the participant moved backward in the list to report a skipped item. Like the overall lag-CRP, the postanticipation lag-CRP is computed for each lag by dividing the number of times a particular lag occurred by the number of times a particular lag was possible. Following previous research, we scored only the first response after anticipation, and all responses prior to the anticipation must have been scored as correct.

When only the first response after an anticipation is included in lag-CRP computations, a − 1 lag represents a fill-in response, and a + 1 lag represents an infill response (although the correspondence is not perfect; see Osth & Dennis, 2015). A fill-in tendency is observed when the lag-CRP of − 1 lag responses is higher than the lag-CRP of + 1 lag responses. An infill tendency is observed when the lag-CRP of + 1 lag responses is higher than − 1 lag responses. It is common to compute an *error ratio* that compares (in the numerator) the likelihood of fill-in responses with (in the denominator) the likelihood of infill responses (Surprenant et al., 2005). Although there is variation in the error ratio from study to study, error ratios around 2.0 are often reported in serial recall tasks (Farrell et al., 2013; Osth & Dennis, 2015).

We compared the lag-CRP of − 1 lag responses and + 1 lag responses in same lists, spun lists, and scrambled lists to determine whether there was a fill-in tendency. We also compared lag-CRPs across list types. Differences in the likelihood of fill-in or infill across list types may provide insight into the mechanisms supporting serial memory. If recall shifts to rely more on item-dependent cues in spun lists than in scrambled lists, then in spun lists infill responses should be more likely and fill-in responses should be less likely.

The circular nature of spun lists also generates a novel prediction about transitions between items at the ends of the list. Imagine a participant had previously recalled the list FABCDE and was later asked to recall the list ABCDEF. In the first list, the participant practiced recalling A after recalling F. If this prior practice influences recall of spun lists—that is, if the participant is using item-dependent cues in these lists—then the participant should be more likely to recall A after F in the latter list (e.g., reporting ABCDFA).^3^ In the current experiments, which all used six-item lists, a transition from the final item to the initial item produced a − 5 lag response. To assess our prediction, we compared the postanticipation lag-CRP of − 5 responses across list types. In spun lists uniquely, such a response is akin to an infill response (+ 1 lag response after an anticipation); both involve a practiced, forward movement to the subsequent list item.

When computing lag-CRP, researchers typically choose to exclude repeat responses: if a participant repeats a previous item (e.g., ABDA) the numerator is not incremented for this response, and denominator is not incremented for any lags that would produce a repeat response. However, because of our scoring method for the postanticipation lag-CRP, excluding repeat responses would make it so that a − 5 lag response could only be scored when participants initiated recall with the final list item and then reported the first list item immediately afterward (e.g., reporting FA to ABCDEF). This pattern of responses is extremely rare, so analysis of − 5 lag responses would have been underpowered (if not impossible) if repeats were removed. We computed lag-CRPs both with repeat responses and without repeat responses, and we report both in the text. However, our statistical analyses were only conducted on lag-CRPs that included repeat responses. The probabilities of fill-in and infill responses were identical regardless of whether repeats were scored. Once again, this was a consequence of our scoring method for postanticipation lag-CRP—if all items before the anticipation error were correct responses, then − 1 lag or + 1 lag response cannot result in a repeat response. Allowing repeats would influence fill-in and infill rates for more liberal scoring methods (e.g., Hurlstone, 2010).

When computing postanticipation lag-CRP, we chose not to score responses given by a participant after they quickly reported the second item in the first position (e.g., BCDEF). We were concerned that some of these trials may have included *false start* responses. False starts are motor errors that can occur when the participant begins responding too early (i.e., typing before the response cue appears) or when the execution of the first keystroke takes abnormally long (i.e., the movement for the first key press is initiated first but finishes after the movement for the second keystroke; Logan, 2018), and they can be detrimental to the estimation of fill-in and infill rates. In the former case, early recall of B is likely to be followed by early recall of C, leading to an inflation of infill responses. In the latter case, early recall of B is likely to be followed by late recall of A, leading to an inflation of fill-in responses. We identified false starts by comparing the response time of the first response to the response times of second responses. First response times are often much slower than second response times (cf. Lindsey & Logan, 2019), so we marked an anticipation in the first position as a false start if its response time was shorter than the 75th percentile of second responses. The 75th percentile cutoff was determined separately for each participant. If an anticipation was marked as a false start, the response after it was not scored in postanticipation lag-CRPs.

Lag-CRPs are typically presented graphically by plotting conditional response probability as a function of lag. When all responses contribute to lag-CRP, the plotted curve peaks at + 1 lags; most responses are correct responses, and prior to an error all correct responses have lags of + 1. When only postanticipation responses contribute to lag-CRP, the curve often peaks at − 1 lag instead, indicating a fill-in tendency (Farrell et al., 2013, but see Solway et al., 2012). We created overall lag-CRP curves, which can be found in Appendix B in the supplemental materials, and postanticipation lag-CRP curves, which can be found in the Results section.

We computed lag-CRPs and postanticipation lag-CRPs for each list type seen by each participant in each experiment. Because all experiments manipulated list type within-subject, participants had lag-CRPs for each list type. Because list length was six for all the analyzed experiments, we computed overall lag-CRPs for lags of − 5 to + 5, and we computed postanticipation lag-CRPs for lags of − 5 through + 4.^4^ To aid comparison to other research, we computed error ratios by dividing the postanticipation lag-CRP of − 1 lag responses by the postanticipation lag-CRP of + 1 lag responses. We additionally computed average fill-in counts and infill counts for each serial position (Appendix B). We used MATLAB (MathWorks, 2021) for all computations.

### Statistical analyses

Statistical analyses were conducted on postanticipation lag-CRPs. Because of their theoretical importance, lags of − 5, − 1, and + 1 were analyzed. To maximize the power of statistical analyses on the archival data, we combined data from the typing experiments of Lindsey and Logan (2019) and separately combined data from the memory experiments of Lindsey and Logan (2021). The typing data from Lindsey and Logan (2019) included 72 participants and the memory data from Lindsey and Logan (2021) included 120 participants. From an experimental design perspective, combining the data in this manner is justifiable because the experiments were structured almost identically and studied identical participant populations. To determine whether combining the data was statistically justifiable, we conducted one-way ANOVA that treated experiment number as a between-subject variable. A separate one-way ANOVA was conducted for each lag (− 5, − 1, and + 1). Participants were excluded from analysis if they had no data for the analyzed lag. The results are shown in Table 2. None of these analyses were significant, so all subsequent analyses of archival data aggregated over experiment. Analyses for the memory archival data and typing archival data were conducted separately. Like the statistical analyses, the lag-CRP curves reported in the Results aggregate over experiment.

**Table 2:** One-way ANOVA to test experiment differences

List type	Lag	*F*	*df*_*B*_	*df*_*W*_	*p*
Memory					
Spun	+ 1	1.02	4	115	0.398
Spun	− 1	0.73	4	114	0.573
Spun	− 5	0.63	4	110	0.645
Scrambled	+ 1	1.40	4	115	0.237
Scrambled	− 1	0.18	4	115	0.951
Scrambled	− 5	1.30	4	112	0.275
Typing					
Spun	+ 1	0.34	2	69	0.716
Spun	− 1	0.21	2	69	0.815
Spun	− 5	1.09	2	63	0.342
Scrambled	+ 1	0.67	2	69	0.514
Scrambled	− 1	0.40	2	69	0.669
Scrambled	− 5	0.77	2	68	0.466

*Note.* Memory data was aggregated from the training portions of Experiments 1–5 in Lindsey and Logan (2021). Typing data was aggregated from the training portions of Experiments 1, 2, and 4 in Lindsey and Logan (2019)

Similarly, for the new experiment we combined data from the two research sites. We had data from 223 participants: 145 participants were from the University of South Alabama, and 78 participants were from the University of North Georgia. Table 3 shows the results of independent-samples *t* tests conducted on postanticipation lag-CRPs, treating research site as a between-subject variable. Only one of the probabilities was significantly different between the two sites: − 1 lag responses in spun lists were less likely at the University of South Alabama. However, we also analyzed lag-CRPs in just the University of South Alabama data and found that the pattern of significant results was identical to the combined data. Given these similarities, combining the data is justified. Analyses for this experiment were conducted separately from the archival data. The lag-CRP curves reported in the Results aggregate over research site.

**Table 3:** Independent-samples *t* tests to test research site differences (USA vs. UNG)

List Type	Lag	*M*_*diff*_	*SE*_*diff*_	*t*	*df*	*p*
Same	+ 1	− 0.013	0.047	− 0.29	201	0.775
Same	− 1	− 0.051	0.048	− 1.06	207	0.291
Same	− 5	0.003	0.005	0.67	102	0.508
Spun	+ 1	− 0.004	0.029	− 0.15	221	0.882
Spun	− 1	− 0.065	0.029	− 2.21	221	0.028
Spun	− 5	0.012	0.029	0.40	196	0.691
Scrambled	+ 1	− 0.040	0.021	− 1.91	219	0.057
Scrambled	− 1	− 0.020	0.028	− 0.73	219	0.465
Scrambled	− 5	0.005	0.022	0.23	202	0.815

*Note.* USA = University of South Alabama; UNG = University of North Georgia. *M*_*diff*_ is the mean of USA minus the mean of UNG

We conducted paired-samples t-tests to assess differences in postanticipation lag-CRPs. These tests were conducted for each data set (archival memory data, archival typing data, and new data). Bayes factors (BF; Rouder et al., 2009) were computed for each *t* test to determine the evidence in favor of a difference. In each list type, we compared the likelihood of − 1 lag responses to the likelihood of + 1 lag responses. This comparison informs whether there was a fill-in tendency or an infill tendency for each list type. We expect there to be a greater likelihood of fill-in (− 1) responses than infill (+ 1) responses in same lists and scrambled lists. We expect there to be a lesser likelihood of fill-in (− 1) responses than infill (+ 1) responses in spun lists.

We additionally assessed differences in specific postanticipation lag-CRPs across list types. Broadly, we were interested in comparing lag-CRPs in same lists to those in spun lists, and lag-CRPs in spun lists to those in scrambled lists. Comparisons of spun and scrambled lists were conducted for all data sets, but same and spun list comparisons were only possible for the new data. We compared the probability of − 1 lag responses across list types to determine if there were differences in the likelihood of fill-in responses. We compared the probability of + 1 lag responses across list types to determine if there were differences in the likelihood of infill responses. We compared the probability of − 5 lag responses across list types to test our prediction about the circularity of spun lists.

## Results

**Fig. 1:**
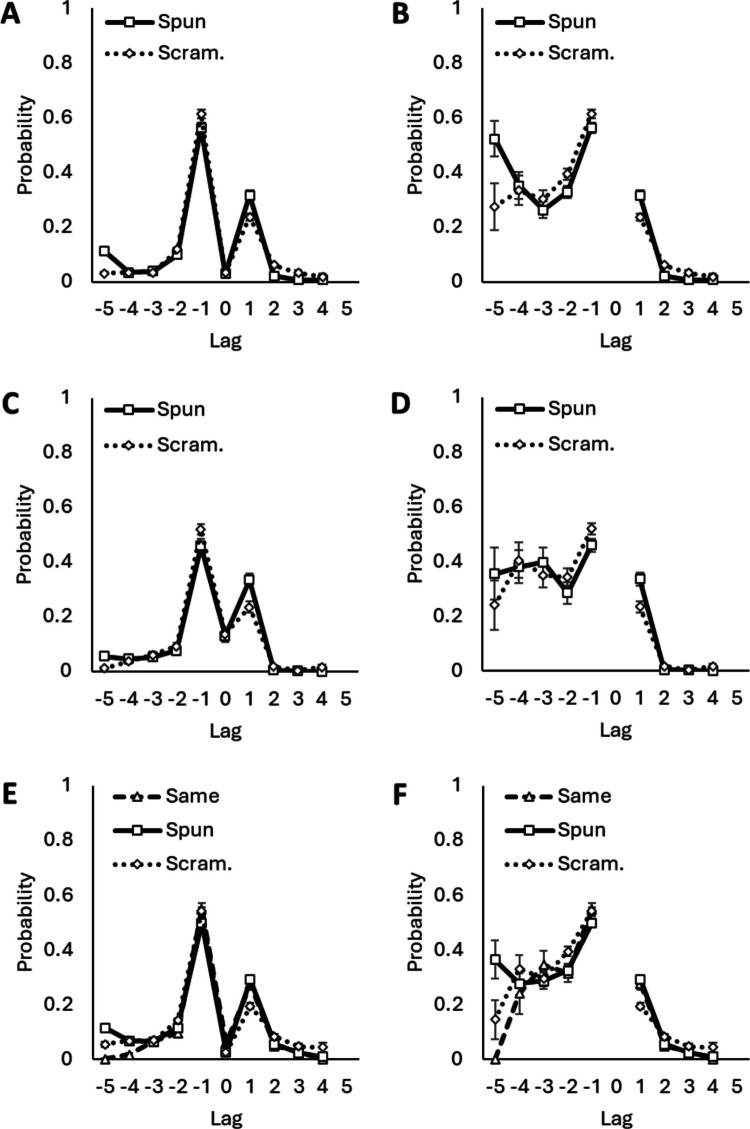
Postanticipation lag-CRP curves for archival memory data (Panels ***A, B***), archival typing data (Panels ***C, D***), and the new experiment (Panels ***E, F***). For curves on the left, the lag-CRPs scored repeat responses. For those on the right, repeat responses were not scored. Data points represent means over participants, and error bars represent standard errors of those means

Postanticipation lag-CRP curves are shown in Fig. 1. Table 4 shows postanticipation lag-CRPs for − 1 lag and + 1 lag responses and error ratios. The results of *t* tests on postanticipation lag-CRPs are shown in Table 5 for the archival data and Table 6 for the new experiment. In all data sets and list types, lag-CRP curves peaked at − 1 lags, and − 1 lag responses were significantly more likely than + 1 lag responses. Consequently, all error ratios were greater than 1.0. However, ratios differed among list types: they were smallest in spun lists and largest in scrambled lists. A fill-in tendency was present in all list types, but the magnitude of this tendency varied.

**Table 4:** Fill-in rate, infill rate, and error ratios

List type	P(Fill-in)	P(Infill)	Error ratio
Memory			
Spun	0.56	0.32	1.81
Scrambled	0.62	0.24	2.64
Typing			
Spun	0.46	0.34	1.38
Scrambled	0.52	0.24	2.23
New Experiment			
Same	0.55	0.28	1.99
Spun	0.50	0.29	1.72
Scrambled	0.54	0.20	2.77

*Note.* Error ratio is P(Fill-In)/P(Infill). Memory data were obtained from the training portions of Experiments 1–5 in Lindsey and Logan (2021). Typing data was obtained from the training portions of Experiments 1, 2, and 4 in Lindsey and Logan (2019). Reported values for archival data average over the analyzed experiments

**Table 5:** Paired-samples *t* tests on postanticipation lag-CRP in the archival data

Test			*M*_*diff*_	*SE*_*diff*_	*t*	*df*	*p*	BF_10_
Fill-in Tendency for Each List Type (Memory)								
Spun − 1	vs	Spun + 1	0.243	0.035	6.95	118	< 0.001	4.25E + 07
Scrambled − 1	vs	Scrambled + 1	0.378	0.025	15.30	119	< 0.001	4.69E + 26
Comparison of Spun and Scrambled Lists (Memory)								
Spun + 1	vs	Scrambled + 1	0.080	0.022	3.71	118	< 0.001	60.2
Spun − 1	vs	Scrambled − 1	− 0.051	0.026	− 2.00	119	0.048	0.688
Spun − 5	vs	Scrambled − 5	0.082	0.017	4.96	112	< 0.001	5,539
Fill-in Tendency for Each List Type (Typing)								
Spun − 1	vs	Spun + 1	0.124	0.039	3.17	71	0.002	12.25
Scrambled − 1	vs	Scrambled + 1	0.285	0.033	8.75	71	< 0.001	1.17E + 10
Comparison of Spun and Scrambled Lists (Typing)								
Spun + 1	vs	Scrambled + 1	0.100	0.026	3.79	71	< 0.001	73.03
Spun − 1	vs	Scrambled − 1	− 0.060	0.024	− 2.49	71	0.015	2.28
Spun − 5	vs	Scrambled − 5	0.048	0.015	3.21	65	0.002	13.38

*Note.* Memory data was aggregated from the training portions of Experiments 1–5 in Lindsey and Logan (2021). Typing data was aggregated from the training portions of Experiments 1, 2, and 4 in Lindsey and Logan (2019). BFs are two-sided Bayes factors (P[alternative]/P[null]).

**Table 6:** Paired-samples *t* tests on postanticipation lag-CRP in the new experiment

Test			*M*_*diff*_	*SE*_*diff*_	*t*	*df*	*p*	BF_10_
Fill-in Tendency for Each List Type								
Same − 1	vs	Same + 1	0.272	0.040	6.80	202	< 0.001	6.56E + 07
Spun − 1	vs	Spun + 1	0.209	0.024	8.72	222	< 0.001	8.25E + 12
Scrambled − 1	vs	Scrambled + 1	0.348	0.020	17.64	220	< 0.001	3.92E + 40
Comparison of Same and Spun Lists								
Same + 1	vs	Spun + 1	− 0.012	0.027	− 0.46	202	0.648	0.09
Spun − 1	vs	Same − 1	0.054	0.025	2.22	208	0.027	0.86
Spun − 5	vs	Same − 5	− 0.095	0.018	− 5.41	91	< 0.001	26,884
Comparison of Spun and Scrambled Lists								
Spun + 1	vs	Scrambled + 1	0.098	0.017	5.88	220	< 0.001	5.91E + 05
Spun − 1	vs	Scrambled − 1	− 0.043	0.018	− 2.41	220	0.017	1.27
Spun − 5	vs	Scrambled − 5	0.055	0.018	2.99	184	0.003	6.13
Comparison of Same and Scrambled Lists								
Same + 1	vs	Spun + 1	0.085	0.024	3.52	200	< 0.001	29.85
Spun − 1	vs	Same − 1	0.001	0.026	0.05	206	0.958	0.08
Spun − 5	vs	Same − 5	− 0.052	0.016	− 3.39	97	0.001	22.14

*Note.* BFs are two-sided Bayes factors (P[alternative]/P[null])

Comparisons of lag-CRPs across list types inform why the fill-in tendency varies. A − 1 lag response was significantly less likely in spun lists than scrambled lists. Fill-in was less likely in spun lists. A + 1 lag response was significantly more likely in spun lists than scrambled lists. Infill was more likely in spun lists.^5^ The combination of these differences produced a weaker fill-in tendency in spun lists. A − 5 lag response was significantly more likely in spun lists than in same or scrambled lists. The circularity of spun lists made − 5 lag responses akin to infill responses, and, like infill responses, these responses were elevated in spun lists.

The fill-in tendency in same lists was weaker than in scrambled lists but stronger than in spun lists. Same lists showed an elevated likelihood of both − 1 lag and + 1 lag responses, and a reduced likelihood of distal lags. A − 1 lag response was significantly more likely in same lists than spun lists, but it was just as likely as a − 1 lag in scrambled lists. Similarly, a + 1 lag response was significantly more likely in same lists than scrambled lists, but just as a likely as a + 1 lag in spun lists. Same lists were like scrambled lists for fill-in responses, but they were like spun lists for infill responses.

## Discussion

We conducted novel analyses of fill-in and infill responses in nine experiments employing the spin list procedure. Based on prior research, we expected to find a fill-in tendency in scrambled lists and same lists, but not in spun lists where the consistent ordering of neighbors promotes the use of chaining-like item-dependent cues. Our expectations were not met: A fill-in tendency was observed in all list types. Recall of all list types was strongly influenced by mechanisms—like a primacy gradient and response suppression (Hurlstone, 2010)—that favor fill-in responses.

However, our analyses revealed important differences in the magnitude of the fill-in tendency among list types: fill-in responses were less likely, and infill responses were more likely in spun lists than in scrambled lists. Like the accuracy advantage for spun lists (Lindsey & Logan, 2021), the elevated likelihood of infill responses is consistent with participants becoming more reliant on item-dependent cues in spun lists. The reduced likelihood of fill-in responses in spun lists suggests a corresponding shift away from mechanisms that support fill-in responses (e.g., weakening of the primacy gradient).

Infill responses were also more likely in same lists than scrambled lists. However, unlike spun lists, the likelihood of fill-in responses was not lower in same lists. Item-dependent cues (that support infill responses) and item-independent cues (that support fill-in responses) seem to operate in tandem. Given the superior performance observed in same lists (Appendix A), serial-order memory benefits when both support performance.

The spin list procedure is methodologically similar to the Hebb repetition learning procedure. Current models of Hebb repetition learning are purely item-independent: Recall improves because participants learn sets of position-based cues that are repeatedly associated with the list items (Burgess & Hitch, 2006) or learn representations of the whole list (Page & Norris, 2009). In the current analyses, the likelihood of infill responses was higher in same lists, which are like repeated lists in Hebb repetition experiments, than in scrambled lists, which are most like nonrepeating control lists in those experiments. Given that infill responses reflect the use of item-dependent cues, our findings suggest that item-dependent cues support Hebb repetition learning as well.^6^ As in serial recall, the best model of Hebb repetition learning likely includes both item-dependent cues and item-independent cues (e.g., position-based associations or whole-list representations).

Although we believe the current analyses inform models of serial-order memory, creating a new model or testing existing models goes beyond the scope of this paper. For this reason, we have been intentionally vague about the nature of item-dependent retrieval cues and other mechanisms used in these tasks. Future modeling efforts will be crucial to answering many questions that remain after the current research. For example, the current results suggest that both item-dependent cues and item-independent cues support serial learning; however, they give little indication of the nature of these cues. Item-dependent cues may be separable from item-independent cues (e.g., the item–item and position–item associations in Burgess & Hitch, 1992). They could instead be the same kind of cue, distinguishable only by the cue’s constituent content or features. That is, the cue may at one time be item-dependent by containing features of previously retrieved items, and at another time be item-independent by not containing these features (e.g., the context cues in Logan, 2021; Logan & Cox, 2023). Attention may provide top-down control over serial order by applying more weight to some features in the cue (e.g., item-dependent information) than others (cf. Logan et al., 2021).

The current paper bolsters the benchmark status of the fill-in tendency, and it provides some novel guidance into how fill-in and infill responses should be used to test models of serial-order memory. Conventionally, researchers have used the presence of the fill-in tendency to adjudicate among models, arguing that its presence rejects pure associative chaining theories and provides strong evidence for a primacy gradient (Page & Norris, 1998). In contrast, the presence of the fill-in tendency was uninformative in the current paper because fill-in responses were more likely than infill responses in all list types. However, there was variation in the magnitude of the fill-in tendency caused by differences in the likelihood of fill-in and infill responses among the list types. Specifically, infill responses, which are conventionally thought to be a hallmark of associative chaining (Henson, 1996), were more likely in spun lists. This finding is consistent with existing evidence of the use of item-dependent cues in spun lists (Lindsey & Logan, 2021). The current results suggest that greater emphasis should be placed on the magnitude of the fill-in tendency—or the separate rates of fill-in and infill responses—and researchers should assess how the magnitude changes across task conditions (e.g., in the spin list learning paradigm). Changes in the magnitude of the fill-in tendency may unveil strategic shifts in the mechanisms or retrieval cues used by the serial-order system (cf. Logan & Cox, 2023; Logie, 2018). In isolation, the fill-in tendency obfuscates the use of item-dependent cues.

Some prior work has addressed variability in the magnitude of the fill-in tendency. Farrell et al. (2013) addressed the influence of list length. The fill-in tendency was weaker in longer lists than shorter ones. Osth and Dennis (2015) manipulated the item set; in one condition list items were sampled with replacement from a small set, and in another they were sampled without replacement from a large set. The fill-in tendency was weaker with large item sets. Surprenant et al. (2005) and Hurlstone (2010) assessed changes in the fill-in tendency as a function of serial position and found that it was relatively stable across serial positions. These analyses provided additional constraints on the mechanisms of serial order (e.g., a model with only a primacy gradient and response suppression incorrectly predicts a larger fill-in tendency at the start of the list; Hurlstone, 2010). We present plots of fill-in and infill responses as a function of serial position in Appendix B.

This prior work entertained and rejected pure associative chaining accounts of the data, because these theories predict an infill tendency rather than a fill-in tendency. A more nuanced account—that item-dependent cues are one of several mechanisms that support serial order—was not considered. The current data likewise reject pure chaining accounts, but they support the more nuanced account; serial order is more reliant upon item-dependent cues when list items have consistent neighbors. The reduction in the fill-in tendency observed by Farrell et al. (2013) and Osth and Dennis (2015) may similarly indicate an increased reliance upon item-dependent cues when recalling long lists created from large item sets. Notably, the experiments analyzed in the current paper all presented short lists sampled with replacement from small sets, so item-dependent cues do not only support long lists (also see Fischer-Baum & McCloskey, 2015).

## Conclusion

The responses made after an anticipation error inform the cognitive mechanisms that underpin serially ordered behavior. However, researchers should be cautious about how they use and interpret these responses. An overall tendency to fill-in skipped items after an anticipation error (a fill-in tendency) is reliably observed in tasks that require serial recall, but the presence (or absence) of the fill-in tendency does not effectively track changes in the serial-order mechanisms that support recall in different task conditions. The magnitude of the fill-in tendency does track these changes. Specifically, increases in the likelihood of infill responses indicate a greater reliance upon item-dependent cues.

## Open practices statement

All task files, data files, analysis files, and analysis scripts are available on the OSF (10.17605/OSF.IO/UBH9Q). The analyses in the paper were not preregistered.

## Supplementary Information

Below is the link to the electronic supplementary material.

Supplementary file1 (DOCX 203 KB)

## Data Availability

Data (Excel files), analysis files (Jamovi files), and task programs (E-Prime files) are available on the OSF (10.17605/OSF.IO/UBH9Q).

## Footnotes

1 Oberauer et al. (2018) categorized the fill-in tendency as a “low-priority” benchmark for general models of working memory. However, they recognized that the effect is well-established in typical serial recall tasks, suggesting that the fill-in tendency is a high-priory benchmark for models of serial recall. The importance of the fill-in tendency as a benchmark of serial order has been made explicit elsewhere (e.g., Osth & Hurlstone, 2023).

2 In Experiment 3, there was a numerical advantage for spun lists that did not reach statistical significance.

3 This prediction is particularly intriguing in the context of the broader serial memory literature. Long-lag responses violate the *locality constraint*—the tendency for items to be reported near to their original positions in the input list (Henson, 1996). As such, these responses are exceedingly rare in typical recall tasks (e.g., Farrell et al., 2013; Osth & Dennis, 2015; Solway et al., 2012).

4 In six-item lists, a lag of + 5 is possible only when the participant transitions from the first list item to the sixth list item. Because reporting the first item can never result in an anticipation error, any transitions from the first item to the sixth item would not contribute to the post-anticipation lag-CRP.

5 A greater prevalence of omission errors has been found to inflate the frequency of infill responses (Osth & Dennis, 2015), so it is noteworthy that there was no difference in the likelihood of omission errors between spun and scrambled lists in the Lindsey and Logan (2021) experiments or the new experiment (Appendix A).

6 Lindsey and Logan (2021) made a similar argument. Anagram lists are thought to be problematic for repetition learning because they generate interference between learned representations of the lists (Page et al., 2013). Although learning was slowed in spun and scrambled lists (both of which are types of anagrams), Lindsey and Logan observed learning in both list types. Learning was superior in spun lists, demonstrating that some kinds of overlap can even be beneficial to repetition learning.

## Code availability

MATLAB code (used to compute conditional response probabilities) is available on the OSF (10.17605/OSF.IO/UBH9Q).
