# Supplementary material for: Item-dependent cues in serial order are tracked by the magnitude (not the presence) of the fill-in tendency
Source: Psychon Bull Rev. 2025 Apr 8;32(5):2146–57. doi: 10.3758/s13423-025-02684-8 (PMC12426145; doi:10.3758/s13423-025-02684-8)
Supplement: Supplementary file 1 — Supplementary file1 (DOCX 203 KB) [file 13423_2025_2684_MOESM1_ESM.docx]

Supplemental Materials

**Appendix A: Methods, Results, and Discussion for the New Experiment**

The new experiment was conducted as part of a larger project investigating individual differences in serial order memory and their relationship to other higher-order abilities such as working memory and novel reasoning ability (e.g. Kane et al., 2004). In this appendix, methodological details and analyses are reported for the serial learning task only. Details for the other tasks will be published in a separate manuscript.

The new experiment used the spin list learning paradigm (Ebenholtz, 1963). The design of the task was based primarily upon the Lindsey and Logan (2021) experiments. Participants saw lists of 6 simultaneously presented letters that they had to report in left-to-right order. Lists repeated multiple times, and we were interested in how performance changed over practice. There were three list types recalled by each participant: same lists (in which order did not change between repetitions), spun lists (in which the order was rotated between repetitions), and scrambled lists (in which order was rearranged pseudo-randomly). See Table 1 in the main text for examples.

We compared response accuracy among the list types to assess the cues that supported serial learning. Better accuracy for same lists than spun lists – the same list advantage – indicates that recall is sensitive to disruptions to serial position and suggests that item-independent cues (specifically position-based cues) support serial learning. Better accuracy for spun lists than scrambled lists – the spun list advantage – indicates that recall is sensitive to disruptions to the identities of neighboring items and suggests that chaining-like item-dependent cues support serial learning. Given prior research (Kahana, Mollison, & Addis, 2010; Lindsey & Logan, 2021), we expected to find both a same list advantage and a spun list advantage.

**Method**

The experiment was conducted at two research sites: University of South Alabama (under the direction of the first author) and University of North Georgia (under the direction of the second author). Ethical approval for this research was obtained at both sites. All participants gave informed consent before beginning the experiment and were compensated with course credit.

***Participants***

A goal of the broader project (of which this experiment was a component) was to model relationships among latent variables using factor analysis and structural equation modeling, so we aimed to recruit at least 200 participants altogether (cf. Shipstead et al., 2014). A total of 223 participants completed the serial learning task – 145 from the University of South Alabama and 78 participants from the University of North Georgia. Participants were between the ages of 18 and 35, reported that they had started learning English before the age of 5, and reported normal or correct-to-normal vision.

Our sample size far exceeded the size of previous spin list learning experiments. For example, Lindsey and Logan (2021) recruited 24 participants per experiment. We had ample statistical power to detect differences among the list types in this experiment.

***Apparatus and Stimuli***

The task was programmed in E-Prime 3.0 (Psychology Software Tools, 2016). The experiment program was run locally at each research site on desktop computers equipped with flatscreen monitors and standard QWERTY keyboards. All text was rendered in black on a white background. Participants used the keyboard to progress through instructions and to type responses for each memory trial. On memory trials, only letter keys and the spacebar were enabled. The identity and timing of each keystroke was recorded by the experiment program.

Participants recalled lists of 6 lowercase letters. To ensure no overlap among the list types, different letters were selected into each list type randomly without replacement. All vowels (including ‘y’) were excluded from selection to reduce the likelihood of producing word-like lists. Random selection occurred for each participant, so different participants recalled different lists, and (across the experiment) each letter was equally likely to be in each list type.

For each list type, we generated 6 lists from the selected letters. For same lists, the 6 lists were identical – the same 6 letters appeared in the same serial positions next to the same neighboring letters. For spun lists, we rotated the 6 letters to produce 6 lists. In these spun lists, each letter occupied each serial position once, but neighboring letters were always the same. For scrambled lists, the letters were rearranged using a 6 x 6 balanced Latin square. In these scrambled lists, each letter occupied each serial position, and the neighboring letters changed for each list.

***Procedure***

Participants completed a 2-hour battery of cognitive tasks. All participants completed the same tasks in the same order: Operation Span, Raven’s Advanced Progressive Matrices, Verbal 3-back, Symmetry Span, Number Series, Visuospatial 3-back, and finally the Serial Learning Task. We first describe the procedure of the new experiment (the Serial Learning Task) in detail. Brief descriptions are offered for the other 6 tasks afterward.

Prior to beginning the Serial Learning Task, we informed participants that they would be memorizing lists of 6 letters, that these 6 letters would be briefly presented on the computer screen simultaneously in a row^[[1]](#footnote-1)^, and that they would be reporting the letters they remember after a brief delay by typing them in left-to-right order. We warned participants that the presentation of the letters would be brief, that they should not begin typing their responses until they are prompted to do so, and that correcting mistakes would not be possible because the backspace key was disabled. Additionally, we asked participants to recall letters as quickly and accurately as possible. Participants were not informed about the different list types.

Each trial began with a centrally presented fixation cross that persisted for 500 ms. The fixation cross was replaced with the list of 6 letters for the current trial, which were also on the screen for 500 ms. After the letters disappeared, there was a 500 ms retention interval during which the screen was blank. The text “Response:” cued the participant to begin typing the letters they could remember. As they typed, their responses were echoed on the screen under the response cue. The participant completed their responses by pressing the spacebar, which ended the current trial and automatically initiated the next trial. The identity and timing of each legal keystroke was recorded by the program.

Each of the 6 same lists, each of the 6 spun lists, and each of the 6 scrambled lists were presented 10 times. We blocked these 18 lists by presentation number: in block 1 each list was presented the first time, in block 2 each was presented the second time, and so on. We randomized the presented order of the 18 lists within each block. There were 10 blocks and 180 total trials. Participants were offered a self-paced break after the completion of the 5th block, but otherwise each block transitioned directly into the next without interruption. The entire task took approximately 15 minutes to complete.

In the Lindsey and Logan (2021) experiments that inspired this task, lists were practiced 40 times each. In the new experiment, we reduced the number of presentations to 10 to make time for an additional list type (same lists) and to lessen the overall time to complete the task. Reducing the number of presentations should not preclude our ability to assess differences among the list types because most of the learning observed in the Lindsey and Logan experiments occurred in the first 10 blocks.

**Operation Span (Foster et al. 2015).** The Operation Span is a “storage-and-processing” task designed to measure a participant’s working memory capacity. It is a serial reconstruction of order task, in which participants see lists of sequentially presented uppercase letters and are asked to report the letters in serial order by clicking (with the computer mouse) them in serial order. Participants must complete a math problem after the presentation of each letter.

**Raven’s Advanced Progressive Matrices (Raven, Raven, & Court, 1998).** Raven’s Advanced Progressive Matrices is a task designed to measure visuospatial reasoning ability. Participants see a 3 x 3 matrix of abstract shapes, and the bottom-right cell of the matrix is blank. Participants must determine which of 8 options best completes the matrix.

**Verbal 3-back (Kirchner, 1958).** The Verbal 3-back task is an “N-back” task, in which a participant responds to a continuous stream of visually presented items by indicating if the current item matched a previously presented item. The “N” specifies the presentation lag between the current item and the previous item. For example, if “N” is 1, the participant must indicate whether the current item was the same item presented 1-back – the item presented just before the current item. In our task, participants remembered letters, common words, and digits in different blocks, and they had to indicate whether each item matched the item 3-back by pressing keys on the computer keyboard.

**Symmetry Span (Foster et al. 2015)**. The Symmetry Span task is a visuospatial analog to the Operation Span task. Participants must remember the locations of sequentially highlighted cells in a 4 x 4 matrix. After each cell is highlighted, the participant must judge whether an image is vertically symmetrical. Participants are asked to report the spatial locations in serial order by clicking cells in a blank matrix.

**Number Series (Thurstone, 1938).** The number series task is a task designed to measure numerical reasoning. Participants a shown a series of numbers with a blank space at the end (e.g., 1, 3, 5, 7, __ ). Participants must determine which of 8 options best completes the series.

**Visuospatial 3-back (Kirchner, 1958).** The Visuospatial 3-back is a visuospatial analog to the Verbal 3-back. Our task was structured identically to the Verbal 3-back, but participants had to remember faces, wingdings, and Japanese kanji instead of letters, words, and digits.

***Analyses***

We scored the accuracy of each response with a strict scoring method: a response was correct if it was an item in the most recently presented list and if its serial position in the response list was the same as its serial position in the presented list. For example, if ABCDEF was presented and ABZCD was reported, only 2 responses would be scored as correct (A and B). We categorized errors into one of three categories. An *intrusion* *error* (or *intrusion*) occurred when the participant reported an item that was not in the most recently presented list. An *order error* (or *misorder*) occurred when the participant recalled an item from the most recent list in the wrong serial position. One or more *omission errors* (or *omissions*) occurred if the participant’s response list was shorter in length than the presented list. In the above example, there was 1 intrusion (Z), 2 misorders (C and D), and 1 omission (the response list was 1 item shorter than the presented list). For each trial, we computed the proportion of correct responses, intrusions, misorders, and omissions by dividing the response counts by list length (6).

We also computed timing measures for each trial. We separately computed the response time for the first keystroke – which we called *initiation time* – and the average of the response times of later keystrokes, which we called *inter-response time*. Initiation times might incorporate the time to encode the response cue, to retrieve the list representation, and to retrieve the first letter in the list, whereas the inter-response times might incorporate the time to retrieve a chunk within the list and to retrieve letters after the first letter. Differences in response times between spun and scrambled lists are generally small or nonsignificant (cf. Lindsey & Logan, 2019, 2021). However, analyzing response time is important to demonstrate that accuracy differences do not reflect a simple speed-accuracy tradeoff.

For each participant, we computed average performance for each list type in each of the 10 presentation blocks. Ther were 6 performance metrics: *error rate* (1 minus the proportion of responses that were correct), *intrusion rate* (the proportion of intrusion errors), *misorder rate* (the proportion of order errors), *omission rate* (the proportion of omission errors), initiation time (average response time for the first keystroke, in ms), and inter-response time (average response time for keystrokes after the first, in ms). These averages were used as the dependent variables in subsequent statistical analyses. Initiation times and inter-response times were only included for correct responses and only if they were less than or equal to 3000 ms.

We first conducted a 2 (research sites) X 3 (list types) X 10 (presentations) mixed factors ANOVA for each of the performance metrics to assess differences in research site. We were most interested in differences among the list types, so we examined the research site X list type interactions to determine whether these differences depended upon where the data were collected. None of these interactions were significant, so we chose to conduct and report simpler analyses that aggregated over research sites.

To assess whether the performance metrics changed over practice, we conducted repeated measures ANOVA for each list type using presentation number as the repeated factor. A significant result suggests that learning of the list improved performance over practice. Participants were excluded from analyses of initiation time or inter-response time if they were missing data for a particular presentation block (because their responses were inaccurate or too long for all pertinent lists in that block).

To compare performance among the list types, we first computed overall error rates, misorder rates, intrusion rates, omission rates, initiation times, and inter-response times for each list type by aggregating over the 10 presentations of each list. To assess whether there was a spun list advantage – that is, more accurate or faster recall of letters in spun lists than scrambled lists – we conducted paired-samples t-tests that compared overall average performance in spun lists to that in scrambled lists (cf. Lindsey & Logan, 2019, 2021). Similarly, to assess whether there was a same list advantage – more accurate or faster recall of letters in same lists than spun lists – we conducted paired-samples t-tests comparing overall same list performance to overall spun list performance.

**Results and Discussion**

In Figure A1, mean error rates, misorder rates, intrusion rates, omission rates, initiation times, and inter-response times are displayed as a function presentation number for each list type. The results of ANOVA that test for learning in each list type are shown in Table A1. Error rates significantly reduced over practice in same lists and spun lists but not in scrambled lists. Misorder rate and intrusion rate significantly decreased in same and spun lists, but not scrambled lists. Omission rate significantly decreased in same lists only – in spun lists there was no significant change, and in scrambled lists there was a small but significant increase. There was evidence of learning in same lists and spun lists, supported by an improved ability to remember the identity of lists items and the order of those items. Initiation times and inter-response times decreased for all list types over practice, hinting that any improvements in accuracy were not a consequence of participants slowing down.


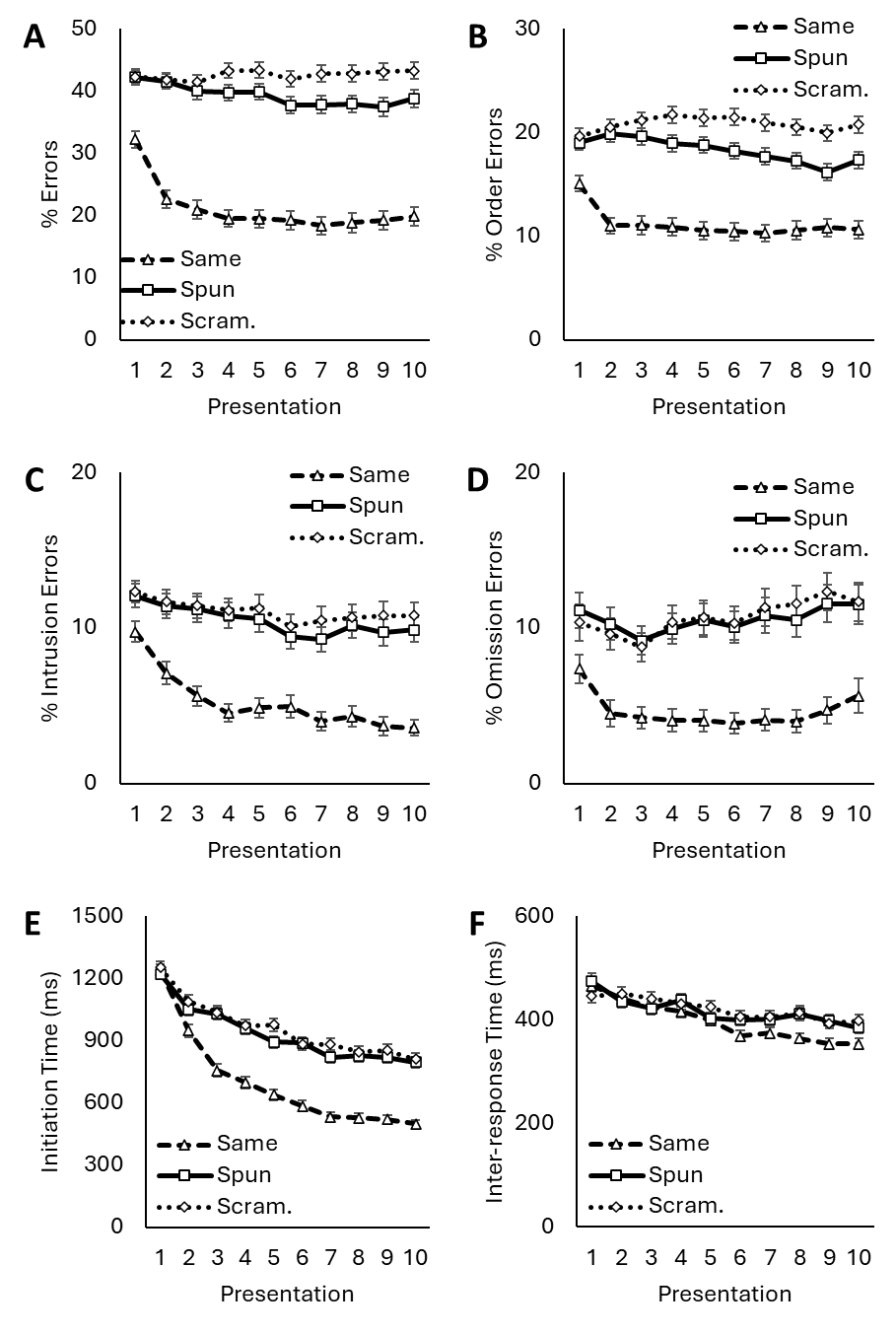


**Figure A1.** Percentages of errors (Panel A), order errors (Panel B), intrusion errors (Panel C), omission errors (Panel D), initiation time (E), and inter-response time (F) for same, spun, and scrambled lists. Data points represent means over participants, and error bars represent standard errors of those means.

| Table A1 | | | | | | |
| --- | --- | --- | --- | --- | --- | --- |
| *ANOVA testing the effect of presentation number.* | | | | | | |
| List Type | *MS_B_* | *df_B_* | *MS_W_* | *df_W_* | *F* | *p* |
| Error Rate | | | | | | |
| Same | 0.376 | 9 | 0.021 | 1998 | 17.74 | <0.001 |
| Spun | 0.061 | 9 | 0.012 | 1998 | 4.95 | <0.001 |
| Scrambled | 0.011 | 9 | 0.010 | 1998 | 1.08 | 0.645 |
| Misorder Rate | | | | | | |
| Same | 0.044 | 9 | 0.010 | 1998 | 4.43 | <0.001 |
| Spun | 0.030 | 9 | 0.008 | 1998 | 3.88 | <0.001 |
| Scrambled | 0.010 | 9 | 0.007 | 1998 | 1.40 | 0.375 |
| Intrusion Rate | | | | | | |
| Same | 0.080 | 9 | 0.004 | 1998 | 21.84 | <0.001 |
| Spun | 0.019 | 9 | 0.005 | 1998 | 3.73 | <0.001 |
| Scrambled | 0.009 | 9 | 0.005 | 1998 | 1.73 | 0.078 |
| Omission Rate | | | | | | |
| Same | 0.027 | 9 | 0.007 | 1998 | 3.71 | <0.001 |
| Spun | 0.012 | 9 | 0.008 | 1998 | 1.50 | 0.143 |
| Scrambled | 0.025 | 9 | 0.008 | 1998 | 3.00 | 0.001 |
| Initiation Time | | | | | | |
| Same | 1.17E+07 | 9 | 6.83E+04 | 1908 | 171.36 | <0.001 |
| Spun | 3.78E+06 | 9 | 6.59E+04 | 1908 | 57.40 | <0.001 |
| Scrambled | 3.75E+06 | 9 | 6.76E+04 | 1899 | 55.50 | <0.001 |
| Inter-response Time | | | | | | |
| Same | 2.26E+05 | 9 | 5.96E+03 | 1035 | 38.00 | <0.001 |
| Spun | 7.35E+04 | 9 | 6.07E+03 | 666 | 12.12 | <0.001 |
| Scrambled | 4.24E+04 | 9 | 6.13E+03 | 603 | 6.92 | <0.001 |
|  |  |  |  |  |  |  |

The results of paired-samples t-tests that compare learning among the list types are shown in Table A2. Error rates were significantly lower in same lists than spun lists, and significantly lower in spun lists than scrambled lists. We observed both a same list advantage (Ebenholtz, 1963) and a spun list advantage (Lindsey & Logan, 2021). All error types were less frequent in same lists than in spun lists. Presenting items in consistent serial positions aided both the recall of an item’s identity and its order in the list. Like in Lindsey and Logan (2021), only misorder errors were less frequent in spun lists than in scrambled lists. Having consistent neighboring items aided only the recall of an item’s order in the list. Response times were not slower for same lists than spun lists, nor were they slower for spun lists than scrambled lists; neither the same list advantage nor the spun list advantage reflects a speed-accuracy tradeoff.

| Table A2 | | | | | | | | |  |
| --- | --- | --- | --- | --- | --- | --- | --- | --- | --- |
| *Paired-samples t-tests comparing performance across list types.* | | | | | | | | |  |
| Test | | | *M_diff_* | *SE_diff_* | *t* | *df* | *p* | BF_10_ |  |
| Error Rate | | | | | | | | |  |
| Same | vs. | Spun | 0.183 | 0.010 | 17.63 | 222 | <0.001 | 5.00E+40 |  |
| Spun | vs. | Scrambled | 0.033 | 0.008 | 3.90 | 222 | <0.001 | 107 |  |
| Misorder Rate | | | | | | | | |  |
| Same | vs. | Spun | 0.072 | 0.007 | 10.72 | 222 | <0.001 | 6.07E+18 |  |
| Spun | vs. | Scrambled | 0.025 | 0.005 | 4.93 | 222 | <0.001 | 6,569 |  |
| Intrusion Rate | | | | | | | | |  |
| Same | vs. | Spun | 0.052 | 0.006 | 8.25 | 222 | <0.001 | 4.10E+11 |  |
| Spun | vs. | Scrambled | 0.006 | 0.006 | 1.03 | 222 | 0.306 | 0.13 |  |
| Omission Rate | | | | | | | | |  |
| Same | vs. | Spun | 0.059 | 0.007 | 8.69 | 222 | <0.001 | 6.88E+12 |  |
| Spun | vs. | Scrambled | 0.001 | 0.004 | 0.33 | 222 | 0.739 | 0.08 |  |
| Initiation Time | | | | | | | | |  |
| Same | vs. | Spun | 229.7 | 21.5 | 10.68 | 222 | <0.001 | 4.42E+18 |  |
| Spun | vs. | Scrambled | 28.9 | 11.7 | 2.47 | 222 | 0.014 | 1.46 |  |
| Inter-response Time | | | | | | | | |  |
| Same | vs. | Spun | 28.3 | 9.3 | 3.05 | 222 | 0.003 | 6.85 |  |
| Spun | vs. | Scrambled | 9.3 | 8.9 | 1.06 | 222 | 0.290 | 0.14 |  |
| The Same vs. Spun comparisons assess whether there is a same list advantage. The Spun vs. Scrambled comparisons assess whether there is a spun list advantage. BFs are two-sided Bayes factors (P[alternative]/P[null]). | | | | | | | | |  |
|  |  |  |  |  |  |  |  |  |  |
|  |  |  |  |  |  |  |  |  |  |
|  |  |  |  |  |  |  |  |  |  |

There was little evidence of learning in scrambled lists in the current experiment – they were recalled faster but not more accurately with practice. This lack of learning is at odds with the experiments of Lindsey and Logan (2021), which consistently found learning in those lists (albeit slower than in spun lists). The improvements in scrambled lists in those experiments were restricted to item errors – participants were less likely to commit intrusion or omission errors with practice. This learning could not have been supported by position-based item-independent cues (because serial position was inconsistent) or item-dependent cues (because items had inconsistent neighbors), so Lindsey and Logan suggested that participants learned the letters in the scrambled set or learned to individuate each scrambled list (leading to less cross-list interference). Compared to the Lindsey and Logan experiments, in the current experiment there were more unique letters to remember, fewer presentations of each list, and more trials between each presentation of a list (because of the inclusion of a third list type). Having more unique letters and fewer and sparser presentations of those letters might have made it more difficult to learn the letters in the scrambled set.

In principle, learning the letter sets could also support spun list recall and same list recall. Like in scrambled lists, this learning could have been disrupted due to the design of the current experiment. However, the use of item-dependent cues was not disrupted in same lists or spun lists (like it was in scrambled lists), and learning was superior in those lists as a result. Similarly, the use of position-based cues was not disrupted in same lists (like it was in spun lists and scrambled lists), and learning was superior in same lists as a result. These differences in learning are evidence that serial order makes use of both position-based item-independent cues and item-dependent cues.

**Appendix B: Supplemental Plots**

**Overall Lag-CRP**

We computed overall lag-CRPs that scored all responses. These lag-CRPs are displayed in Figure B1. All of the curves peak at +1 lag responses, indicating an overwhelming tendency to (correctly) move one position forward in the list. The probability of +1 lag responses was higher in spun lists than scrambled lists, echoing the spun list recall accuracy advantage observed by Lindsey and Logan (2021). The probability of +1 lags was also higher in same lists than spun lists, echoing the same list advantage observed by Kahana, Mollison, and Addis (2010). Lag 0 responses, which are immediate repetitions of an item, were rare. Generally, conditional recall probability was higher at shorter lags than at longer lags, so data sets showed evidence of the locality constraint. The likelihood of long lags was lower in same lists than spun or scrambled lists, so the locality constraint was stronger in same lists. Importantly, the likelihood of lag -5 responses was elevated for spun lists in both all sets. This violation of the locality constraint is consistent with the use of item-dependent cues; these cues bridge the edges of the circular spun lists and make -5 lag responses like +1 lag responses (cf. Raskin & Cook, 1937).


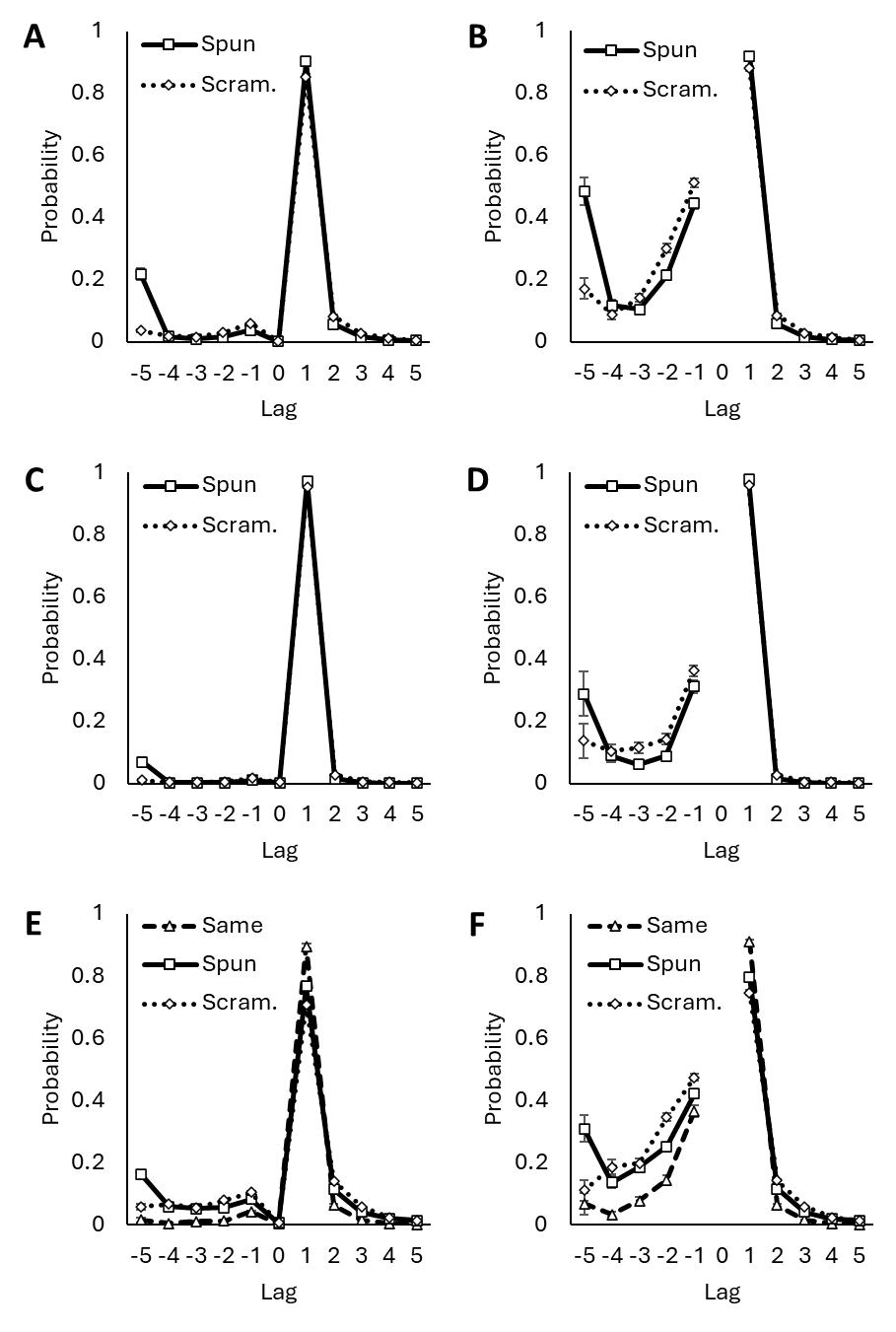


**Figure B1.** Overall lag-CRP curves for archival memory data (Panels A, B), archival typing data (Panels C, D), and the new experiment (Panels E, F). For curves on the left, the lag-CRPs scored repeat responses. For those on the right, repeat responses were not scored. Data points represent means over participants, and error bars represent standard errors of those means.

**Accuracy Serial Position Curves**

For each data set (archival memory data, archival typing data, and the new experiment), we computed average accuracy for each list type as a function of serial position. For archival data, we averaged over the participants in each experiment, then averaged the experiment data. Serial position curves are presented in Figure B2. Broadly, the curves show the strong primacy and weak recency effects that are typical of verbal serial recall (Hurlstone, Hitch, & Baddeley, 2014). The serial position curve for spun lists in the archival typing data is an intriguing exception – it shows a primacy effect but no recency effect. The same list advantage and spun list advantage are evident in the serial position curves; overall, accuracy was lower for spun lists than same lists, and lower for scrambled lists than spun lists. The same list advantage was only present for serial positions 2-6, and the spun list advantage was only present for positions 3-6. The order manipulations primarily influenced an individual’s ability to remember letters at the more difficult serial positions in the middle or end of the list.


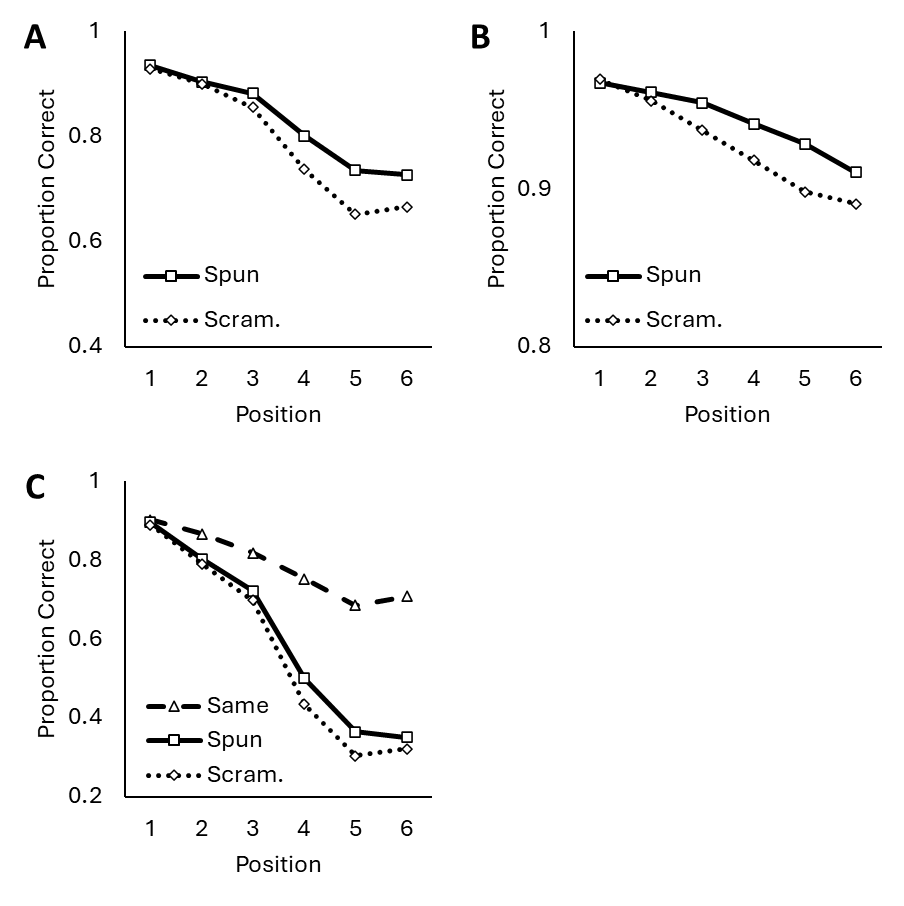


**Figure B2.** Serial position curves for archival memory data (Panel A), archival typing data (Panel B), and the new experiment (Panel C). Data points represent means over participants.

The high accuracy of responses at early positions and the propensity to make fill-in responses both indicate that earlier list items are generally more accessible in memory than later list items. The accessibility of early list items is thought to arise, in part, because of a primacy gradient that activates early list items more strongly than later list items (Henson, 1996). Fill-in rates were lower in spun lists than scrambled lists, despite little difference in recall accuracy between the list types at early list positions. Likewise, high accuracy for early list items is expected even in the absence of a primacy gradient (e.g., Farrell & Lewandowsky, 2004). The rate of fill-in responses may better track the strength of the primacy gradient.

**Fill-in and Infill Counts by Position**

Prior research (e.g., Hurlstone, 2010; Surprenant et al. 2005) analyzed the frequencies of fill-in responses and infill responses over serial positions. This research found that fill-in responses were more frequent than infill responses at all serial positions. At no point in the list was there a shift from a fill-in tendency to an infill tendency (or vice-versa), so there was no apparent shift in the mechanisms used to retrieve items (e.g., from a primacy gradient to associative chaining) in the middle of the list. Hurlstone (2010) additionally found that, under similar scoring procedures as the current paper (which he called conservative scoring), the ratios of fill-in responses to in-fill responses showed a recency effect for end-of-list serial positions but were otherwise stable. Hurlstone demonstrated that a combination of several mechanisms (i.e., a primacy gradient, response suppression, position markers, and an end marker that boosts activation of the final list item) were required to adequately capture changes in the ratio over serial position.

For each participant, we computed the number of fill-in and infill responses at each serial position. Because we presented 6 item lists, fill-in responses could have occurred at positions 2-6, and infill responses could have occurred at positions 2-5. We averaged the response frequencies across participants in each of the three data sets (archival memory data, archival typing data, and new experiment data). The average frequencies are presented in Figure B3. We chose not to present the ratio of fill-in responses to infill responses in these plots like Hurlstone (2010). The ratios obscure changes in each response type; for example, a lowered ratio might reflect a decrease in fill-in responses, an increase in infill responses, or both. As demonstrated in this paper, it is important to interpret fill-in and infill responses separately.

Comparing the left and right panels of Figure B3 reveals that, like Surprenant et al. (2005) and Hurlstone (2010), there was no apparent switch from a fill-in tendency to an infill tendency at any serial position. Infill responses were less frequent than fill-in responses for nearly every serial position, even in spun lists where recall seemed to rely more on item-dependent cues. The presence of a fill-in tendency rejects pure associative chaining accounts.


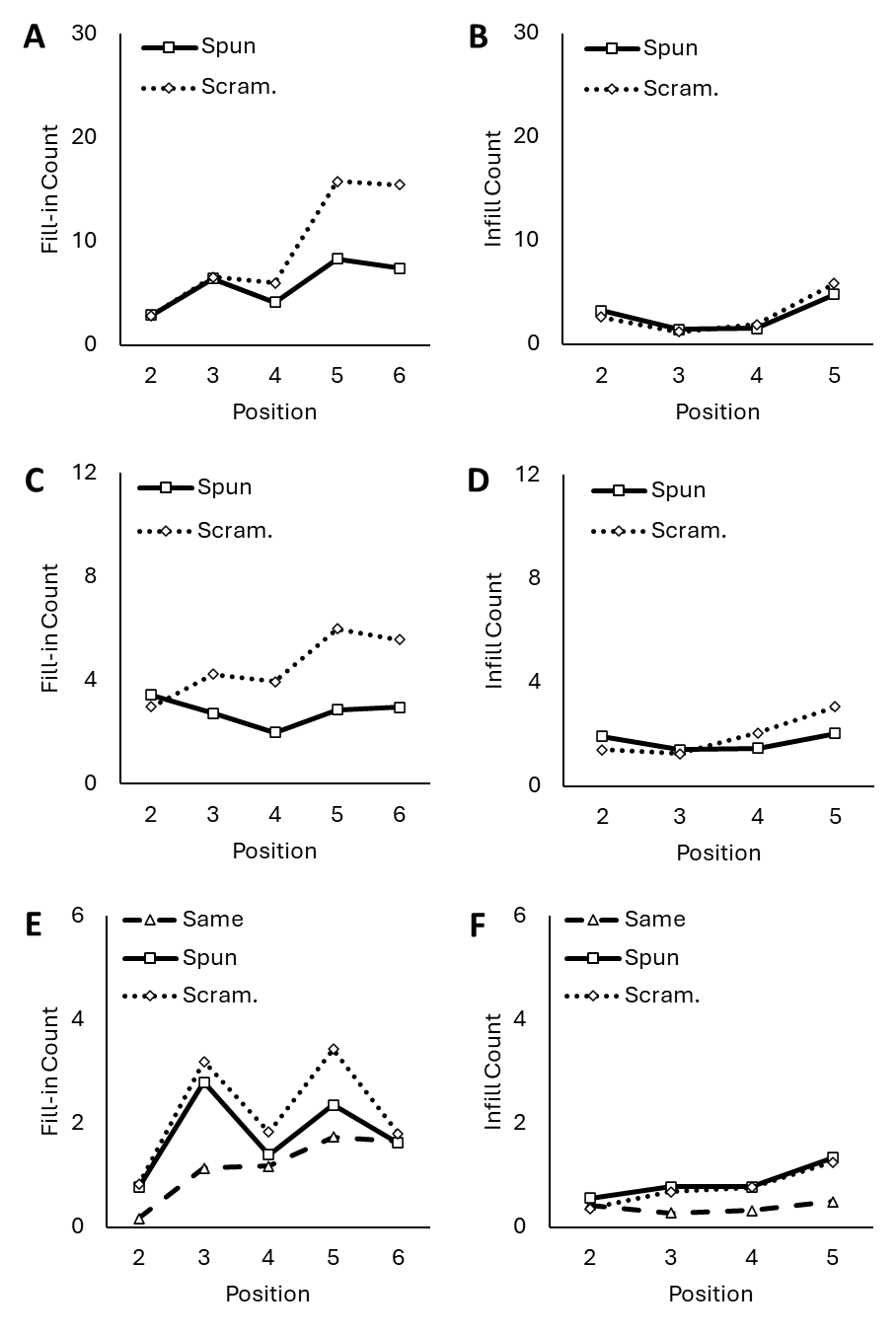


**Figure B3.** Frequencies of fill-in responses and infill responses in archival memory data (Panels A, B), archival typing data (Panels C, D), and the new experiment (Panels E, F). Graphs on the left show fill-in data, and graphs on the right show infill data. Data points represent means over participants.

Unlike lag-CRPs (presented in the main text), raw frequencies do not account for the number of opportunities an individual has to make a fill-in or infill response. In the current paper, this is problematic for two reasons. First, as illustrated in the serial position curves in the previous section, more errors were committed at later serial positions than at early ones. A priori, one would expect to see a greater number of fill-in and infill responses at later list positions simply because there are more opportunities to make these responses. Second, as illustrated in Appendix A, the frequency of order errors differed among the list types. A priori, one would expect fewer fill-in and infill responses in same lists, which had the fewest number of errors, and more fill-in and infill responses in scrambled lists, which had the highest number of errors. We describe the trends in the frequency data below to facilitate comparisons between the current work and prior work. However, we purposefully avoid tying any trends in the frequencies to serial order mechanisms (and we encourage the reader to be cautious of making these connections in the current data).

Generally, fill-in responses were infrequent at early list positions and became more frequent toward the end of the lists. Barring a few exceptions, we found strong recency effects and no primacy effects for fill-in responses. For spun lists in the typing experiments, we found a weak primacy and weak recency effect. For spun and scrambled lists in the new experiment, there were peaks in the curve at positions 3 and 5. These peaks may be a consequence of participants spontaneously grouping the lists, which can distort patterns of fill-in and infill responses (Farrell, 2012). There are smaller scallops in the curve at identical locations in spun lists, implying that the same grouping strategy might have been applied to all list types. Fill-in responses were most common in scrambled lists and least common in same lists, and differences in the counts were most apparent at the end of the list.

Infill responses were largely stable over serial position. Generally, they were a little more likely at the end of the list, so we found weak recency effects. In some curves (e.g., spun and scrambled lists in the archival memory data), we found even weaker primacy effects. Infill responses were least frequent in same lists, but they occurred roughly equally often in spun and scrambled lists.

1. In serial recall tasks, items are typically presented sequentially. Simultaneous presentation alters encoding strategies, leading to superior memory of the list (Ordonez Magro et al. 2022). However, similar serial order phenomena (e.g., transposition gradients) are obtained with both presentation methods, and prior research using the spin list procedure found that presentation method had little influence on the difference between list types (Lindsey & Logan, 2021). We chose simultaneous presentation to be consistent with methods in the archival experiments. [↑](#footnote-ref-1)
